# Xue-Jie-San restricts ferroptosis in Crohn’s disease via inhibiting FGL1/NF-κB/STAT3 positive feedback loop

**DOI:** 10.3389/fphar.2023.1148770

**Published:** 2023-04-19

**Authors:** Ying Gao, Zhaozheng Zhang, Jun Du, Xiao Yang, Xiaopeng Wang, Ke Wen, Xueliang Sun

**Affiliations:** Department of Colorectal Surgery, Suzhou TCM Hospital Affiliated to Nanjing University of Chinese Medicine, Suzhou, China

**Keywords:** Xue-Jie-San, crohn’s disease, ferroptosis, fibrinogen-like protein 1, colitis

## Abstract

Crohn’s disease (CD) is an incurable inflammatory bowel disease due to unclear etiology and pathogenesis. Accumulating evidences have shown the harmful role of ferroptosis in CD onset and development. Additionally, fibrinogen-like protein 1 (FGL1) has been verified to be a potential therapeutic target of CD. Xue-Jie-San (XJS) is an effective prescription for treating CD. However, its therapeutic mechanism has not been fully elucidated. This study aimed to determine whether XJS alleviating CD via regulating ferroptosis and FGL1 expression. A colitis rat model was induced by 2,4,6-trinitrobenzene sulfonic acid and treated with XJS. The disease activity indices of the colitis rats were scored. Histopathological damage was assessed using HE staining. ELISA was performed to examine inflammatory cytokines. Transmission electron microscopy was utilized to observe ultrastructure changes in intestinal epithelial cells (IECs). Iron load was evaluated by examining iron concentrations, the expressions of FPN, FTH and FTL. Lipid peroxidation was investigated through detecting the levels of ROS, 4-HNE, MDA and PTGS2. Furthermore, the SLC7A11/GSH/GPX4 antioxidant system and FGL1/NF-κB/STAT3 signaling pathway were examined. The results showed that colitis was dramatically ameliorated in the XJS-treated rats as evidenced by relief of clinical symptoms and histopathological damages, downregulation of pro-inflammatory cytokines IL-6, IL-17 and TNF-α, and upregulation of anti-inflammatory cytokine IL-10. Furthermore, XJS administration led to ferroptosis inhibition in IECs by reducing iron overload and lipid peroxidation. Mechanistically, XJS enhanced the SLC7A11/GSH/GPX4 antioxidant system negatively regulated by the FGL1/NF-κB/STAT3 positive feedback loop. In conclusion, XJS might restrain ferroptosis in IECs to ameliorate experimental colitis by inhibition of FGL1/NF-κB/STAT3 positive feedback loop.

## Introduction

Crohn’s disease (CD), as a subtype of inflammatory bowel disease (IBD), is a chronic, disabling intestinal disorder with multiple complications. Due to unclear etiology and pathogenesis, therapeutic approaches of CD exhibit diversity. In the biologic era, CD treatment has derived a great achievement. Currently, infliximab, a tumor necrosis factor α (TNF-α) antagonist, remains the mainstream drug for CD management. In patients with biologic naïve CD, infliximab achieves a primary clinical response rate of 58.4% and a 1-year clinical remission rate of 60% (Narula et al., 2022; Wong et al., 2022). Ustekinumab is a novel biological agent for CD treatment by targeting interleukin (IL)-12/IL-23. Although ustekinumab achieves a similar clinical efficacy as infliximab in treating biologic naïve CD, its endoscopic remission rate is lower (Narula et al., 2022; Wong et al., 2022). Combination therapy with an immunomodulator agent is utilized to improve the clinical efficacy of biological agents. However, combination therapy may significantly increase the risk of severe complications. An effective complementary therapy is desired to enhance CD treatment. Traditional herbal therapy has displayed a prominent role in CD management (Torres et al., 2019).

Xue-Jie-San (XJS), a traditional Chinese herbal compound consisting of Dragon’s blood (Resina draconis) and Myrrh (Myrrha), is effective on ameliorating the clinical symptoms of CD patients and promoting healing of intestinal ulcers (Xu et al., 2022). Thus, it is of great value to further explore the underlying mechanisms of XJS on alleviating CD. Based on bioinformatic analysis, our previous study has demonstrated that XJS may reduce lipid peroxidation (LPO) in the colon tissues of CD rat model induced by 2,4,6-trinitrobenzene sulfonic acid (TNBS) (Hong et al., 2022). Excessive LPO is a vital trigger of ferroptosis. As a novel form of regulated cell death, ferroptosis is characterized by iron overload and accumulation of lethal levels of lipid hydroperoxides (Wu et al., 2021). Nowadays, ferroptosis is confirmed to be one of the several important causative factors of IBD. Intestinal epithelial cells (IECs) of CD patients highly express pro-ferroptosis genes like acyl-CoA synthetase long-chain family member 4 (ACSL4), RPL8 and MTDH, followed by reduced expressions of anti-ferroptosis genes, such as glutathione peroxidase 4 (GPX4), ferritin heavy chain (FTH) and zinc finger protein 36 (ZFP36) (Xu et al., 2021; Zhang et al., 2022). Ferroptosis-induced death of IECs destructs the integrity of intestinal epithelial barrier, causes microorganism invasion into intestinal tissue, and consequently aggravates CD progression (Tang et al., 2021). Ferroptosis inhibitor Ferrostatin-1 can mitigate TNBS-induced CD-like colitis (Xu et al., 2021). Apart from classic ferroptosis inhibitors, multiple Chinese herbal extracts exhibit the ability of suppressing ferroptosis to ameliorate IBD-like experimental colitis, such as astragalus polysaccharide, curculigoside, and Shaoyao Decoction (Wang et al., 2020; Chen et al., 2021; Li et al., 2022). A recent study has revealed the anti-ferroptosis action of Loureirin C extracted from Dragon’s blood, which indicates the colitis-protective effect of XJS by acting as a ferroptotic inhibitor (Liu et al., 2023).

Our previous study has screened out fibrinogen-like protein 1 (FGL1) as a potential biomarker and therapeutic target of CD by proteomics (Sun et al., 2021). FGL1, also known as hepassocin or hepatocyte-derived fibrinogen-related protein 1 (HFREP1), is a hepatocyte-secreted acute phase reactant involving in a variety of inflammatory diseases (Liu and Ukomadu, 2008; Liu et al., 2020; Sun et al., 2021; Liu et al., 2022; Sui et al., 2022). Functionally, FGL1 belongs to a fibrinogen superfamily, and participates in the formation of a plasma clot through non-covalently binding to fibrin and fibrinogen (Rijken et al., 2006). Given the efficacy of XJS on promoting blood circulation and removing blood stasis, FGL1 may be its potential target. Additionally, FGL1 can also regulate lipid metabolism, which plays a vital role in the process of ferroptotic cell death (Liu et al., 2022).

Additionally, our previous study also demonstrates that FGL1 aggravates inflammatory response in IECs by activating nuclear factor-kappaB (NF-κB) (Sun et al., 2021). Activated NF-κB transcriptionally upregulates IL-6 expression, which thereby intensifies the phosphorylation of signal transducer and activator of transcription-3 (STAT3) through binding to gp130. As a downstream signaling, FGL1 expression is further enhanced by activated STAT3 (Cheng et al., 2018). Collectively, FGL1, NF-κB and STAT3 constitute a positive feedback loop. Nowadays, it has been validated that NF-κB and STAT3 are regulators of ferroptosis. The available evidences show benefits of NF-κB and STAT3 on ferroptosis inhibition in IECs (Xu et al., 2020; Huang et al., 2022). However, in the conventional cognition, NF-κB and STAT3 are critical pro-inflammatory signals and overactivated in the damaged intestine of CD patients (Vavricka et al., 2018). Targeting inhibition of NF-κB and STAT3 has become an important strategy for the treatment of CD-like experimental colitis (Zhao et al., 2021; Zhang et al., 2022). Therefore, treating CD by inhibition of NF-κB- and STAT3-regulated ferroptosis is controversial, and the action of NF-κB and STAT3 to regulate ferroptosis remains unclear. In the present study, we hypothesized that XJS might ameliorate CD via repression of ferroptosis regulated by the FGL1/NF-κB/STAT3 positive feedback loop. The findings showed that XJS ameliorated TNBS-induced experimental colitis via suppression of ferroptosis positively regulated by the FGL1/NF-κB/STAT3 positive feedback loop.

## Materials and methods

### Preparation of XJS

XJS, consisting of two medicinal herbs [Dragon’s blood (Resina draconis) 6 g and Myrrh (Myrrha) 6 g], was prepared using ethanol extraction (Table 1) (Hong et al., 2022). Briefly, after Dragon’s blood and Myrrh were crushed and mixed, the mixture was extracted with 95% ethanol under heating reflux condition for 2 h. Then, ethanol in the solutions derived from 3 times extraction was volatilized in water bath. Finally, the extracts were dried in vacuum for reserve.

**TABLE 1:** Composition of XJS prescription.

Chinese name	English name	Latin name	Plant name	Part used	Producer	Weight (g)
Xue Jie	Dragon’s blood	Resina draconis	Dracaena cochinchinensis (Lour.) S. C. Chen	Resin	Yunnan, China	6
Mo Yao	Myrrh	Myrrha	Commiphora myrrha Engl.	Resin	Guangxi, China	6

### LC-MS analysis

The Thermo Vanquish UHPLC (Thermo Fisher Scientific, United States) and Q-Exactive HF (Thermo Fisher Scientific, United States) were used for LC-MS analysis of XJS. After XJS samples were crushed and cooled with low-temperature ultrasound, they were centrifuge at 12,000 × *g* for 10 min at 4°C to collect the supernatant. The diluted supernatant was added with internal standard and filtered through a 0.22 μm PTFE filter. The separations of the samples were implemented on the Zorbax Eclipse C18 column (2.1 mm × 100 mm, 1.8 μm). The column temperature was 30°C and the flow rate was 0.3 mL/min. The mobile phase included 0.1% formic acid (A) and pure acetonitrile (B). The gradient elution procedure was as follows: 0–2 min, 5% B; 2–7 min, 30% B; 7–14 min, 78% B; 14–20 min, 95% B; 20–21 min, 5% B. The MS analysis was performed in positive and negative ion modes. In the two modes, the electrospray voltage was 3.5 KV and the capillary temperature was 330 °C. The range of full mass scan was 100–1,050 m/z. The experimental data was collected by Compound Discoverer 3.3.

### Animal experiment

The animal experiments were reviewed and approved by the Ethics Committee of Suzhou TCM Hospital Affiliated to Nanjing University of Chinese Medicine (No. 2021-LDP-055). Male Sprague-Dawley (SD) rats (specific-pathogen free (SPF) grade, weight 240–310 g) were obtained from the Experimental Animal Center of Nanjing University of Chinese Medicine [animal license No. SYXK (Su) 2018–0049]. All rats were housed in a temperature-controlled room (20–25°C) with constant humidity (45%–55%) and a 12 h light/dark cycle. The rats fed and drank unrestrictedly.

The rats were randomly divided into 5 groups: (1) Control group, (2) Model group, (3) XJS low-dose (XJS-L) group, (4) XJS high-dose (XJS-H) group, and (5) Sulfasalazine (SASP) group. Six rats were included in each group. The CD rat model was induced by TNBS (Sigma, United States) referring to a prior literature (Peterson et al., 2000). Briefly, following 24 h of fasting, a single dose of 50 mg TNBS dissolving in 50% ethanol (v/v) was infused into the lumen of rat colon through a 3.5 F catheter under anesthesia. Insertion depth of the catheter was 8 cm proximal to the anus. After infusion, the rats were kept in the Trendelenburg position for 1 min to ensure the solution contacting with the intestinal mucosa. On the day of colitis induced, the rats in the control group received a saline enema instead of TNBS. On the 3rd-14th day, the rats in XJS-L and XJS-H groups received once-daily intragastric administration of XJS (1.26 g/kg and 2.52 g/kg respectively, weight ratio between crude drug and rat). The rats in SASP group were treated with SASP (100 mg/kg, Fuda, China), who in the other two groups were treated with an equal volume of normal saline. All rats were euthanized on the 15th day and the colon tissues were collected for analysis.

### Evaluation of colitis symptoms

Body weight, fecal occult blood, and stool consistency of the rats were recorded to evaluate the colitis symptoms respectively on day 0, day 2, day 8 and day 14. Scores of the three symptoms were calculated according to the previously reported criteria (Table 2) (Li et al., 2021). The ratio of body weight change (%) was counted as the following formula: (current body weight—initial body weight)/initial body weight. The disease activity index (DAI) was scored as the following formula: DAI = (total scores of body weight loss, fecal occult blood, and stool consistency)/3. In addition, the entire colon length from the anus to the end of the cecum was immediately measured after the rats were sacrificed.

**TABLE 2:** Disease activity index scale.

Score	Body weight loss (%)	Stool consistency	Fecal occult blood
0	< 1	Normal	Negtive
1	1–5	Soft	Weakly positive (+)
2	5–10	Mushy	Positive (++)
3	10–15	Porridge	Strongly positive (+++)
4	> 15	Watery	Visible bleeding

### Histopathological assessment

The colon samples were fixed in 4% formaldehyde solution for 24 h. Then, they were dehydrated with ethanol and embedded in paraffin. Finally, the paraffin block was serially sliced into 4 μm thickness for hematoxylin and eosin (HE) staining. The morphological changes of the colon tissues were observed using an optical microscope. Histological scores were calculated referring to a prior study (Cheng et al., 2022).

### Enzyme-linked immunosorbent assay (ELISA)

The colon samples were homogenized and centrifuged at 3,000 × *g* for 20 min to collect the supernatant. Levels of IL-6, IL-10, IL-17 and TNF-α were examined using ELISA kits (Jingmei Biotechnology, Jiangsu, China) according to the manufacturer’s protocol.

### Transmission electron microscopy (TEM)

The colon tissues were segmented into 1 mm^3^ sections and fixed with 1% osmium acid for 2 h at room temperature. The samples were dehydrated with ethanol, permeabilized, and embedded in Luveak 812. The ultrathin sections were stained successively by 2% uranyl acetate and 2.6% lead citrate, and examined via TEM.

### Iron assay

Iron levels in the colon tissues were determined by iron assay kit (Leagene Biotechnology, Beijing, China) according to the manufacturer’s instructions. Briefly, the colon samples were homogenized and centrifuged at 5,000 × *g* for 15 min to collect the supernatant for examination. Total protein concentration was quantitated by bicinchoninic acid (BCA) assay (Beyotime Biotechnology, Shanghai, China). Subsequently, 75 μL supernatant was incubated with 200 μL Fe assay buffer for 10 min at 37°C. The absorbance value was measured at 562 nm using a microplate reader.

### Malondialdehyde (MDA) measurement

The MDA levels in the colon tissues were measured by MDA assay kit (Solarbio, Beijing, China) according to the manufacturer’s instructions. In brief, the colon samples were homogenized and centrifuged at 8,000 × *g* and 4°C for 10 min to collect the supernatant for measurement. Next, 200 μL supernatant was added into a 96-well microplate, and the absorbance values at 532 nm and 600 nm were respectively measured by a microplate reader.

### Assessment of reduced glutathione (GSH)

The levels of reduced GSH in the colon tissues were examined by reduced GSH assay kit (Solarbio, Beijing, China) according to the manufacturer’s instructions. The absorbance value was measured at 412 nm by a microplate reader.

### Quantitative real time polymerase chain reaction (qRT-PCR) analysis

Total RNA was extracted from the colon tissues using TRIzol reagent (Solarbio, Beijing, China) according to the manufacturer’s protocol. Then, cDNA was reversely transcribed from total RNA with PrimeScript RT Master Mix (Yeasen Biotechnology, Shanghai, China). A qPCR was performed using SYBR Green Master Mix (Yeasen Biotechnology, Shanghai, China). The mRNA levels of ferroportin (FPN), FTH, ferritin light chain (FTL), prostaglandin endoperoxide synthase 2 (PTGS2), GPX4 and FGL1 were quantified and GAPDH was used as the control. The relative gene expressions were normalized to GAPDH and calculated with 2^−ΔΔCT^. The primer sequences were listed in Table 3.

**TABLE 3:** Primer sequences used in qRT-PCR.

Gene	Forward	Reverse
FPN	TAG​TAT​TGA​TTT​CAG​TCT​CCT​T	CTT​TCA​TCT​GTA​ACT​TCC​TTT​T
FTH	TCT​GTC​CAT​GTC​TTG​TTA​TTT​T	AGT​CAT​CAC​GGT​CAG​GTT​TCT​T
FTL	AGA​TGA​ATG​GGG​TAA​AAC​CTG​CT	TCG​AGA​AGT​CAC​AGA​GAT​GAG​GG
PTGS2	GAT​GAC​TGC​CCA​ACT​CCC​A	TGA​ACC​CAG​GTC​CTC​GCT​T
GPX4	GCC​GTC​TGA​GCC​GCT​TAC​T	AGT​GCC​CGT​CGA​TGT​CCT​T
FGL1	CAA​AAT​CAA​ACC​TCT​TCA​GAG​CC	GTT​TCA​TAG​TCA​TTC​CAA​CCC​CT
GAPDH	CCA​TCA​CTG​CCA​CTC​AGA​AGA​C	GAT​ACA​TTG​GGG​GTA​GGA​ACA​C

### Western blot analysis

The colon samples were homogenized, lysed with RIPA lysis buffer, and centrifuged at 12,000 × *g* and 4 C for 10 min to collect the supernatant. Total protein concentration was quantitated by BCA assay kit (Beyotime Biotechnology, Shanghai, China). The proteins were separated in sodium dodecyl sulfate-polyacrylamide gel electrophoresis (SDS–PAGE) and transferred onto polyvinylidene fluoride (PVDF) membranes. The membranes were blocked with 5% skim milk for 2 h at room temperature, and subsequently incubated with primary antibodies against FPN (ZY-1986Ab, Zeye Biotechnology, Shanghai, China), FTH (DF6278, Affinity, Australia), FTL (10727-1-AP, Proteintech, United States), PTGS2 (66351-1-Ig, Proteintech, United States), GPX4 (67763-1-Ig, Proteintech, United States), solute carrier family 7 member 11 (SLC7A11; 26864-1-AP, Proteintech, United States), STAT3 (10253-2-AP, Proteintech, United States), phosphorylated STAT3 (p-STAT3; AF3293, Affinity, Australia), NF-κB (p65) (80979-1-RR, Proteintech, United States), p-p65 (AF 2006, Affinity, Australia), FGL1 (DF13014, Affinity, Australia), GAPDH (60004-1-Ig, Proteintech, United States) overnight at 4°C. After wash 3 times, the membranes were incubated with horseradish peroxidase (HRP)-conjugated secondary antibodies (A0192 and A0208, Beyotime Biotechnology, Shanghai, China) for 1.5 h at room temperature. Finally, the protein signals were visualized by ECL solution and quantified by ImageJ software.

### Immunohistochemical staining

The colon samples were fixed in 4% formaldehyde solution for 24 h, dehydrated with ethanol, embedded in paraffin, and eventually sectioned into 4 μm thickness. The sections were deparaffinized in xylene, rehydrated with ethanol, and heated for antigen retrieval. After the endogenous peroxidase activity was blocked by 3% hydrogen peroxide, the sections were incubated in 5% bovine serum albumin to block non-specific immunoglobulin binding. Subsequently, the samples were incubated with primary antibody against 4-hydroxynoneal (4-HNE; ab48506, Abcam, United Kingdom) at 4°C overnight. Following wash, the sections were incubated with an HRP-conjugated secondary antibody (A0192, Beyotime Biotechnology, Shanghai, China) for 50 min at room temperature. Finally, the sections were stained with diaminobenzidine kit (KGP1045-100, KeyGEN Biotechnology, Nanjing, China) and counterstained with haematoxylin. The slides were observed using a light microscope. ImageJ software was used to calculate the positive staining area.

### Reactive oxygen species (ROS) assay

ROS levels in the colon tissues were determined using 2,7-dichlorodi-hydrofluorescein diacetate (DCFH-DA) reactive oxygen fluorescent probe (G1706-100T, Servicebio, Wuhan, China) according to the manufacturer’s instructions. Images were captured under a fluorescent microscope. ImageJ software was used to measure the fluorescence intensity.

### Statistical analysis

All data were shown as mean ± standard deviation (mean ± SD). Statistical differences among multiple groups were analyzed by GraphPad Prism 9.4 (La Jolla, CA, United States) using the one-way ANOVA analysis. Statistical significance was defined as *p* < 0.05.

## Results

### Identified chemical components of XJS

**FIGURE 1:**
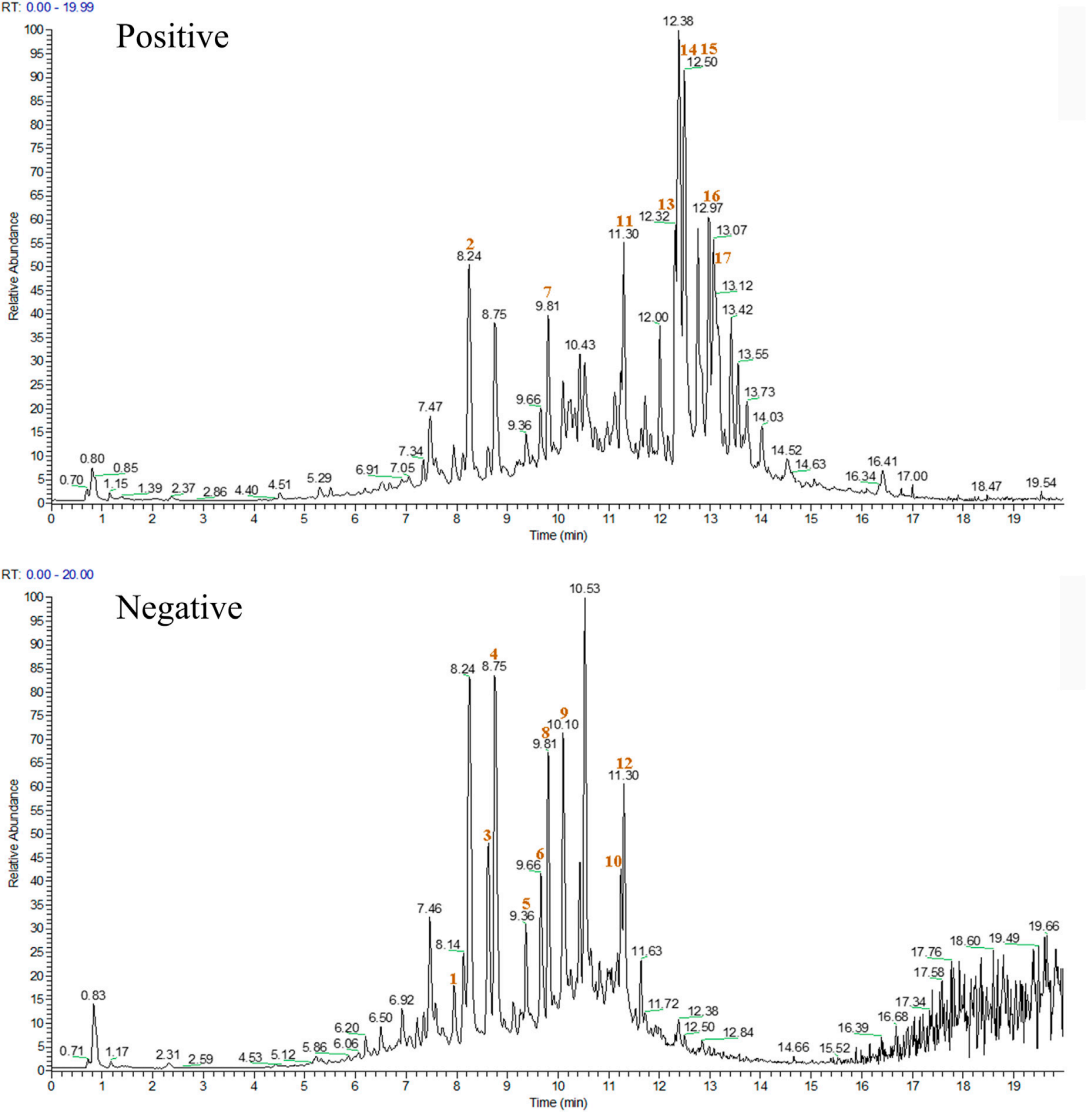
The chromatogram of XJS in positive and negative ion modes. The red number indicated the location of the 17 chemical components.

The chemical components of XJS were isolated and detected by HPLC-MS/MS (Figure 1). The data found that the ethanol extract contained 17 components with a relative percentage of more than 1%, belonging to linear 1,3-diarylpropanoids, benzene and substituted derivatives, organooxygen compounds, naphthofurans, prenol lipids, isoflavonoids, aurone flavonoids (Table 4). Based on a literature search through the PubMed database, four chemical components probably played an important role in treating CD-like experimental colitis, including Daidzein, Loureirin B, Dehydrocostus lactone, and Atractylenolide I (Shen et al., 2019; Sun et al., 2020; Qin et al., 2021; Yuan et al., 2022).

**TABLE 4:** Identified chemical components of XJS.

No.	RT (min)	ESI	Molecular weight	Molecular formula	Error (ppm)	Component	CAS
1	7.949	[M-H]^-^	254.05829	C15H10O4	1.51	Daidzein	486–66–8
2	8.24	[M + H]^+^	300.09984	C17H16O5	0.22	2′,4′-Dihydroxy-3,4-dimethoxychalcone	4315–88–2
3	8.621	[M-H]^−^	302.0798	C16H14O6	2.51	2,6,3′-Trihydroxy-4′-methoxy-2-benzylcoumaranone	NA
4	8.758	[M-H]^−^	288.10044	C16H16O5	2.32	4,2′,4′-Trihydroxy-3-methoxydihydrochalcone	NA
5	9.366	[M-H]^−^	270.08977	C16H14O4	2.07	Isoliquiritigenin 4-methyl ether	13,351–10–5
6	9.661	[M-H]^−^	272.10542	C16H16 O4	2.05	4′-O-Methyldavidigenin	65,428–04–8
7	9.801	[M + H]^+^	166.06317	C9H10O3	1.08	o-Veratraldehyde	86–51–1
8	9.807	[M-H]^-^	302.11596	C17H18O5	1.77	Lusianin	168,180–11–8
9	10.101	[M-H]^−^	286.08473	C16H14O5	2.12	Homobutein	21,583–31–3
10	11.243	[M-H]^−^	286.12123	C17H18O4	2.51	Loureirin A	119,425–89–7
11	11.299	[M + H]^+^	180.07877	C10H12O3	0.69	3′,4′-Dimethoxyacetophenone	1,131–62–0
12	11.306	[M-H]^−^	316.13191	C18H20O5	2.63	Loureirin B	119,425–90–0
13	12.31	[M + H]^+^	288.13633	C17H20O4	0.59	Methyl 5-[(4-tert-butylphenoxy)methyl]-2-furoate	NA
14	12.405	[M + H]^+^	228.11525	C15H16O2	0.96	Bisphenol A	80–05–7
15	12.494	[M + H]^+^	230.13083	C15H18O2	0.66	Lindenenol	26,146–27–0
16	12.977	[M + H]^+^	230.13086	C15H18O2	0.8	Dehydrocostus lactone	477–43–0
17	13.111	[M + H]^+^	230.1308	C15H18O2	0.52	Atractylenolide I	73,069–13–3

NA, not available.

### XJS mitigated the symptoms of TNBS-induced experimental colitis

**FIGURE 2:**
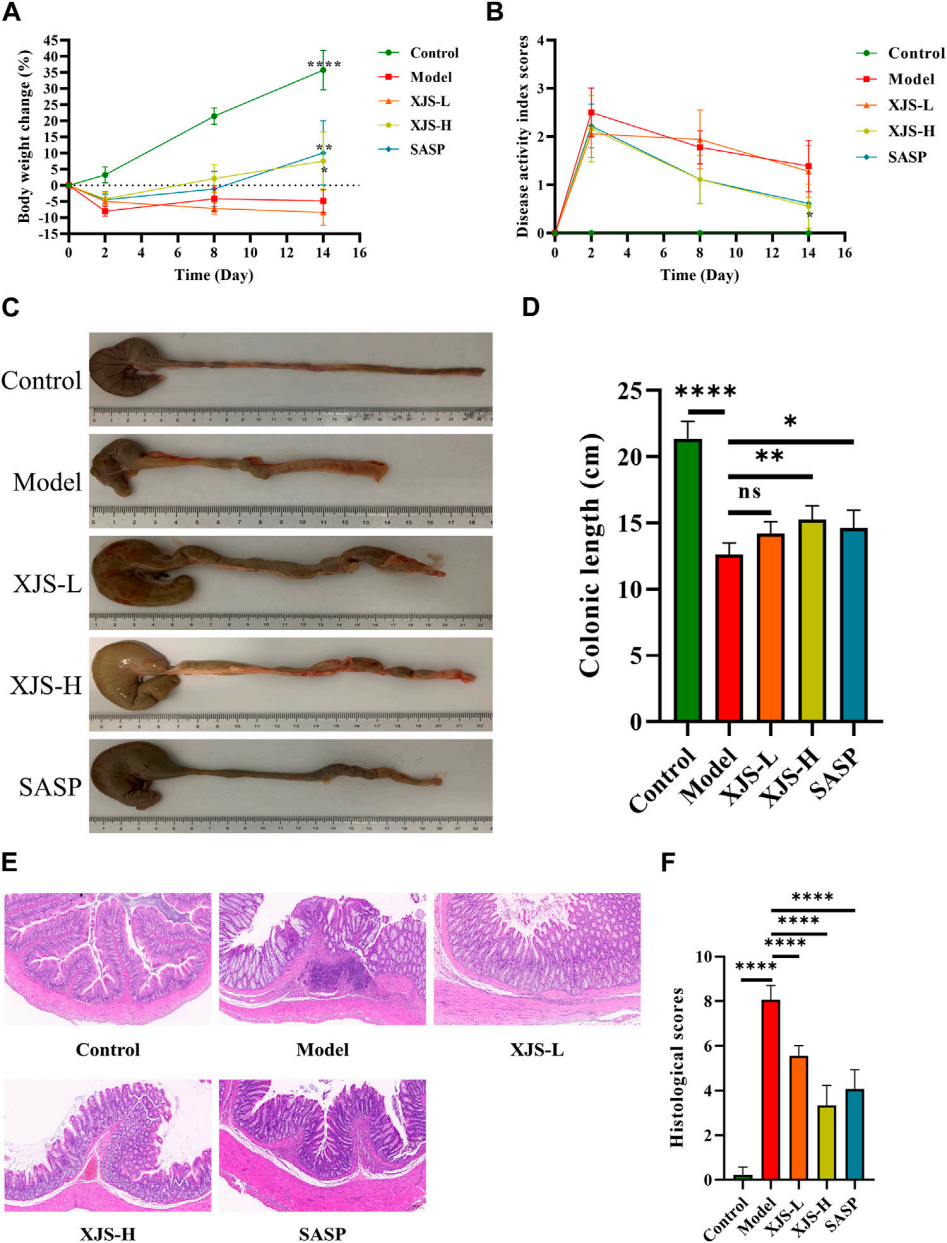
XJS alleviated TNBS-induced colitis. **(A)** The body weight changes on day2, day 8 and day 14 (*n* = 6). **(B)** The DAI scores recorded on day 0, day 2, day 8 and day 14 (*n* = 6). **(C, D)** Colon length (*n* = 6). **(E)** Representative pictures of HE staining (*n* = 6). **(F)** Histological scores (*n* = 6). ^*^ *p* < 0.05, ^**^ *p* < 0.01, ^****^ *p* < 0.0001, ns = no significance.

To assess the effects of XJS on treating CD, a TNBS-induced colitis model was established and treated with XJS. After TNBS induction, rats presented as body weight loss, stool consistency decrease, and fecal blood, which were alleviated by XJS and SASP administration. In detail, the ratio of body weight change had no statistical difference between groups on the 2nd and 8th day. On the 14th day, the body weight of the rats in the control group increased 35.8%, which decreased 4.8% in the model group. The difference between the two groups had statistical significance (*p* < 0.0001). Compared to the model group, low-dose XJS treatment did not significantly change the body weight. However, high-dose XJS and SASP administration respectively increased the body weight of 7.6% and 10.1%, which were markedly higher than that of the model group (*p* = 0.0397, *p* = 0.0093) (Figure 2A). The DAI scores of TNBS-challenged rats dramatically increased. On the 2nd and 8th day, the DAI scores of the XJS-L, XJS-H and SASP groups had no statistical difference compared to that of the model group. On the 14th day, only high-dose XJS treatment led to a significantly lower DAI score (0.56 ± 0.46) than that (1.39 ± 0.53) of the model group (*p* = 0.0439) (Figure 2B). Colon length shortening is an important feature of colitis. Comparing with the control group, the colon length of the rats in the model group markedly decreased [(21.4 ± 1.3) cm vs. (12.6 ± 0.9) cm, *p* < 0.0001]. High-dose XJS and SASP treatment made the colon dramatically longer than that of the model group ((15.2 ± 1.1) cm vs. (12.6 ± 0.9) cm, *p* = 0.0037; (14.6 ± 1.4) cm vs. (12.6 ± 0.9) cm, *p* = 0.0359) (Figures 2C, D).

### XJS alleviated histological damages of the colon tissues

Histopathological damages in the colon tissues were assessed by HE staining. Compared to the control group, pathological presentation in the model group was characterized by inflammatory cell infiltration, submucosal edema, crypt structure destruction, ulcer formation, which contributed to a higher histological score (0.22 ± 0.35 vs. 8.06 ± 0.65, *p* < 0.0001). XJS and SASP treatment led to a reduction in the amounts of inflammatory cells and improvement of submucosal edema, crypt structure destruction and mucosa ulcer (Figure 2E). The histological scores of the XJS-L, XJS-H and SASP groups were significantly lower than that of the model group (5.56 ± 0.46 vs. 8.06 ± 0.65, *p* < 0.0001; 3.33 ± 0.90 vs. 8.06 ± 0.65, *p* < 0.0001; 4.06 ± 0.88 vs. 8.06 ± 0.65, *p* < 0.0001) (Figure 2F). The effect of XJS on treating colitis was in a dose-dependent manner.

### XJS ameliorated inflammatory status in the colon tissues of CD rats

**FIGURE 3:**
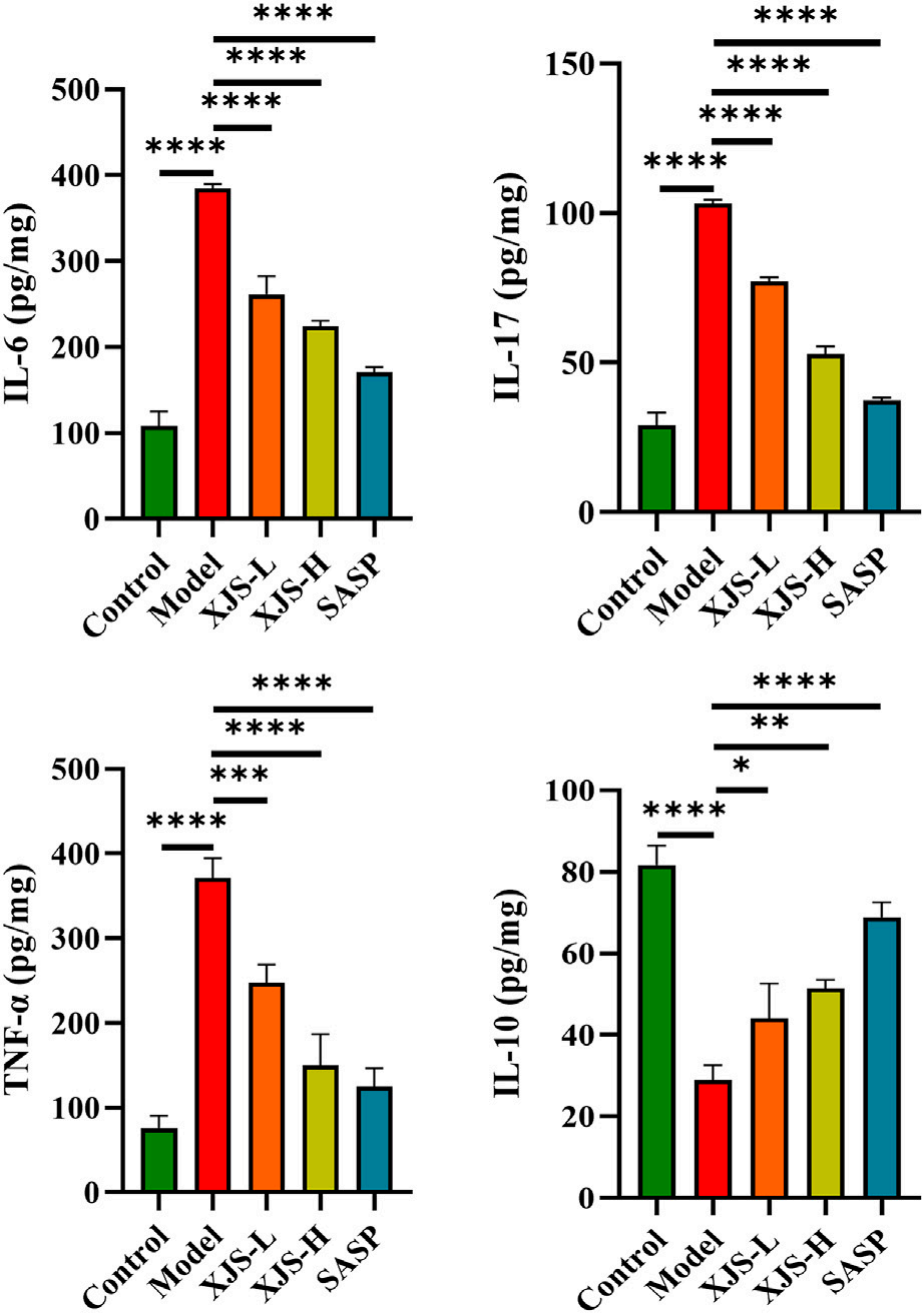
The expression levels of inflammatory cytokines IL-6, IL-17, TNF-α, and IL-10 in the colon tissues (*n* = 3). ^*^ *p* < 0.05, ^**^ *p* < 0.01, ^***^ *p* < 0.001, ^****^ *p* < 0.0001.

To assess the influence of XJS on inflammatory status, the levels of pro-inflammatory cytokines (IL-6, IL-17, TNF-α) and anti-inflammatory cytokine (IL-10) in the colon tissues were detected by ELISA. Compared to the control group, the model group exhibited upregulated levels of IL-6, IL-17 and TNF-α and a reduced level of IL-10, which suggested an enhanced inflammatory response. XJS and SASP administration obviously reversed the expressions of the abovementioned cytokines, presenting an inhibition effect on the inflammatory responses in the colon tissues of the CD rats (Figure 3).

### XJS prevented ferroptosis in IECs

**FIGURE 4:**
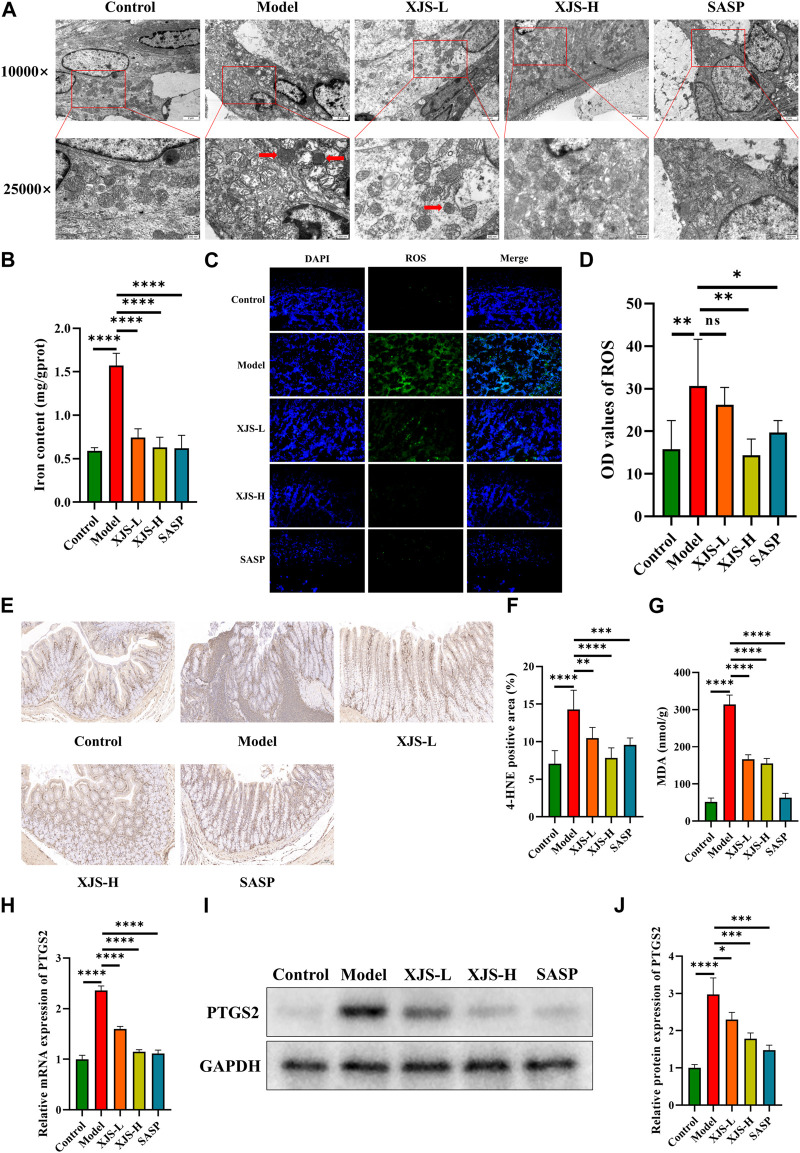
XJS inhibited ferroptosis in intestinal epithelial cells. **(A)** Shrunken mitochondria captured by TEM (red arrows) (*n* = 5). **(B)** Iron levels (*n* = 6). **(C, D)** ROS levels detected by fluorescent probe (original magnification, ×200; *n* = 6). **(E, F)** The expressions of 4-HNE examined by immunohistochemical staining (*n* = 6). **(G)** MDA levels (*n* = 3). **(H)** The mRNA expression levels of PTGS2 (*n* = 6). **(I, J)** The protein expressions of PTGS2 (*n* = 3). ^*^ *p* < 0.05, ^**^ *p* < 0.01, ^***^ *p* < 0.001, ^****^ *p* < 0.0001, ns = no significance.

Given the protective function of intestinal epithelial barrier against inflammatory stimulators in the colon lumen, ultrastructure changes in IECs were observed using TEM. Obviously shrunken mitochondria and reduction or absence of mitochondrial spine were found after TNBS challenge, which were critical features of ferroptosis (Figure 4A). In order to further demonstrate the occurrence of ferroptosis, iron levels in the colon tissues were firstly determined. Results showed an obviously increased level of iron in the model group comparing with the control group (Figure 4B). Furthermore, LPO was assessed. Markedly elevated levels of ROS and LPO products (4-HNE, MDA) were verified in the model group (Figures 4C–G). Additionally, the mRNA and protein expressions of PTGS2, an important marker of ferroptosis, were also upregulated in the model group (Figures 4H–J). Above results together demonstrated that ferroptosis did occur in TNBS-induced colitis. XJS and SASP treatment significantly reduced the amounts of shrunken mitochondria and the levels of iron, ROS, 4-HNE, MDA and PTGS2, which displayed an anti-ferroptosis function of XJS (Figures 4A–J).

### XJS relieved iron overload by regulation of iron transport and storage

**FIGURE 5:**
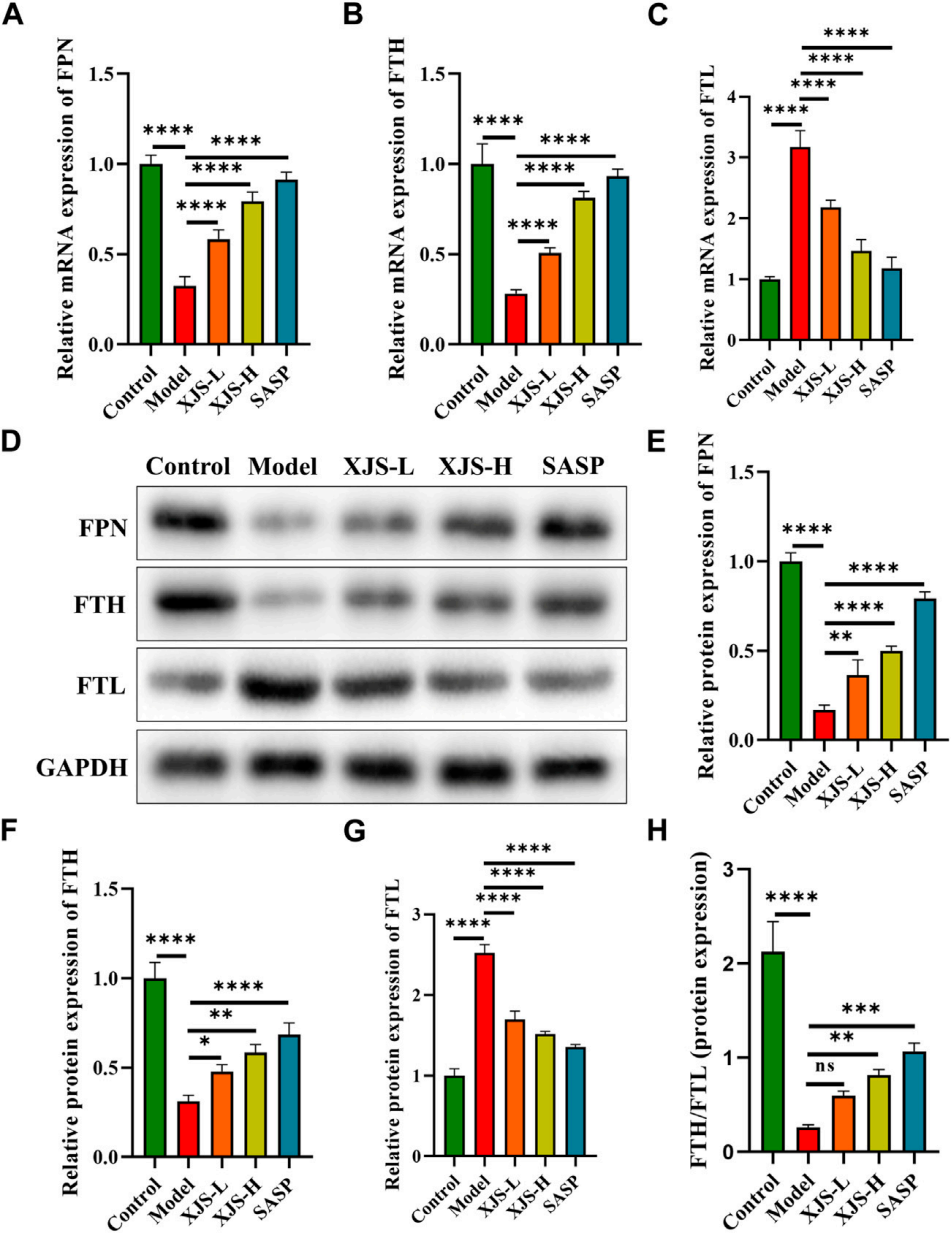
XJS regulated the transport and storage of iron in colon tissues of CD rats. **(A–C)** The mRNA expressions of FPN, FTH and FTL (*n* = 6). **(D–G)** The protein expressions of FPN, FTH and FTL (*n* = 3). **(H)** The ratio of FTH/FTL protein expression (*n* = 3). ^*^ *p* < 0.05, ^**^ *p* < 0.01, ^***^ *p* < 0.001, ^****^ *p* < 0.0001, ns = no significance.

Intracellular iron homeostasis was maintained by transmembrane iron export and ferritin storage. To elucidate the mechanism of XJS on repressing iron overload, the levels of the sole transmembrane iron exporter FPN, iron storage protein FTH and FTL were determined. Compared to the control group, the model group exhibited a decrease in the mRNA and protein expressions of FPN, which were reversed by XJS and SASP treatment in a dose-dependent manner (Figures 5A, D, E). This finding confirmed that XJS enhanced the transmembrane export of intracellular iron. On the other hand, TNBS challenge downregulated the FTH expression and upregulated the FTL expression at the levels of transcription and protein, which were reversed by XJS and SASP treatment (Figures 5B–D, F, G). Further analysis found an elevated ratio of FTH/FTL protein expression after high-dose XJS and SASP administration (Figure 5H). These results suggested that XJS increased intracellular iron storage by regulating ferritin expression.

### XJS reduced LPO by enhancing the activity of the SLC7A11/GSH/GPX4 antioxidant axis

**FIGURE 6:**
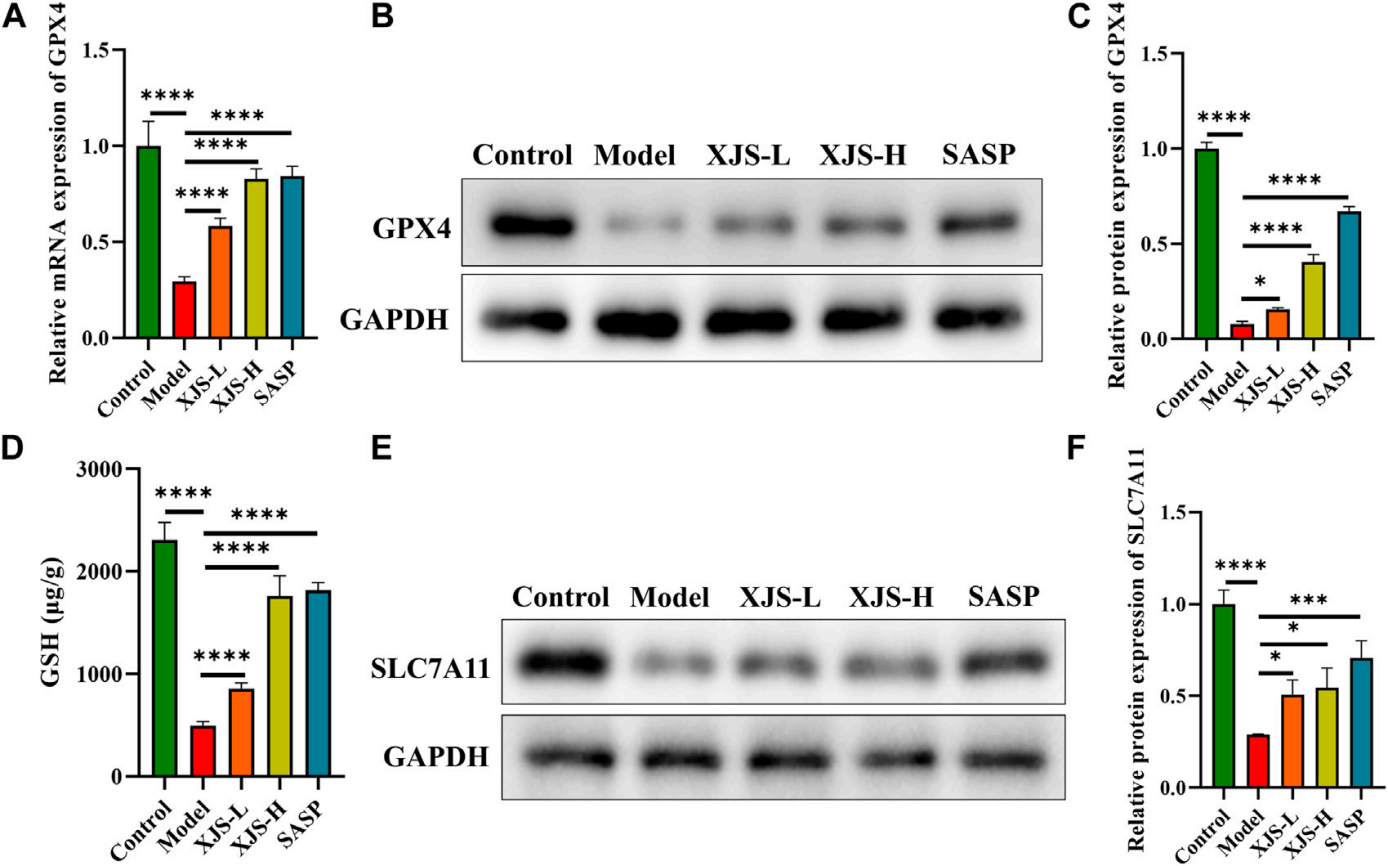
XJS enhanced SLC7A11/GSH/GPX4 antioxidant axis. **(A)** The mRNA expressions of GPX4 (*n* = 6). **(B, C)** The protein expressions of GPX4 (n = 3). **(D)** The levels of GSH (*n* = 3). **(E, F)** The protein expressions of SLC7A11 (*n* = 3). ^*^ *p* < 0.05, ^***^ *p* < 0.001, ^****^ *p* < 0.0001.

To reveal the mechanism of XJS on inhibiting LPO, the classical antioxidant axis of SLC7A11/GSH/GPX4 was examined. GPX4 is a crucial inhibitor of LPO. Compared to the control group, the mRNA and protein expressions of GPX4 were obviously downregulated in the model group. XJS and SASP treatment significantly upregulated the expressions of GPX4 at the levels of transcription and protein (Figures 6A–C). Functionally, GPX4 can utilize reduced GSH to reduce toxic lipid hydroperoxides into non-toxic lipid alcohols. The GSH level in the model group was lower than that of the control group. XJS and SASP treatment markedly increased the levels of GSH (Figure 6D). Biosynthesis of GSH is controlled by SLC7A11 that transports extracellular cystine into cells. Furthermore, the protein expressions of SLC7A11 were determined by western blot. Compared to the control group, the SLC7A11 expression in the model group was significantly downregulated, which was reversed by XJS and SASP treatment (Figures 6E, F). Above results demonstrated that XJS enhanced the activity of the SLC7A11/GSH/GPX4 antioxidant axis to inhibit LPO.

### XJS suppressed the FGL1/NF-κB/STAT3 positive feedback loop

**FIGURE 7:**
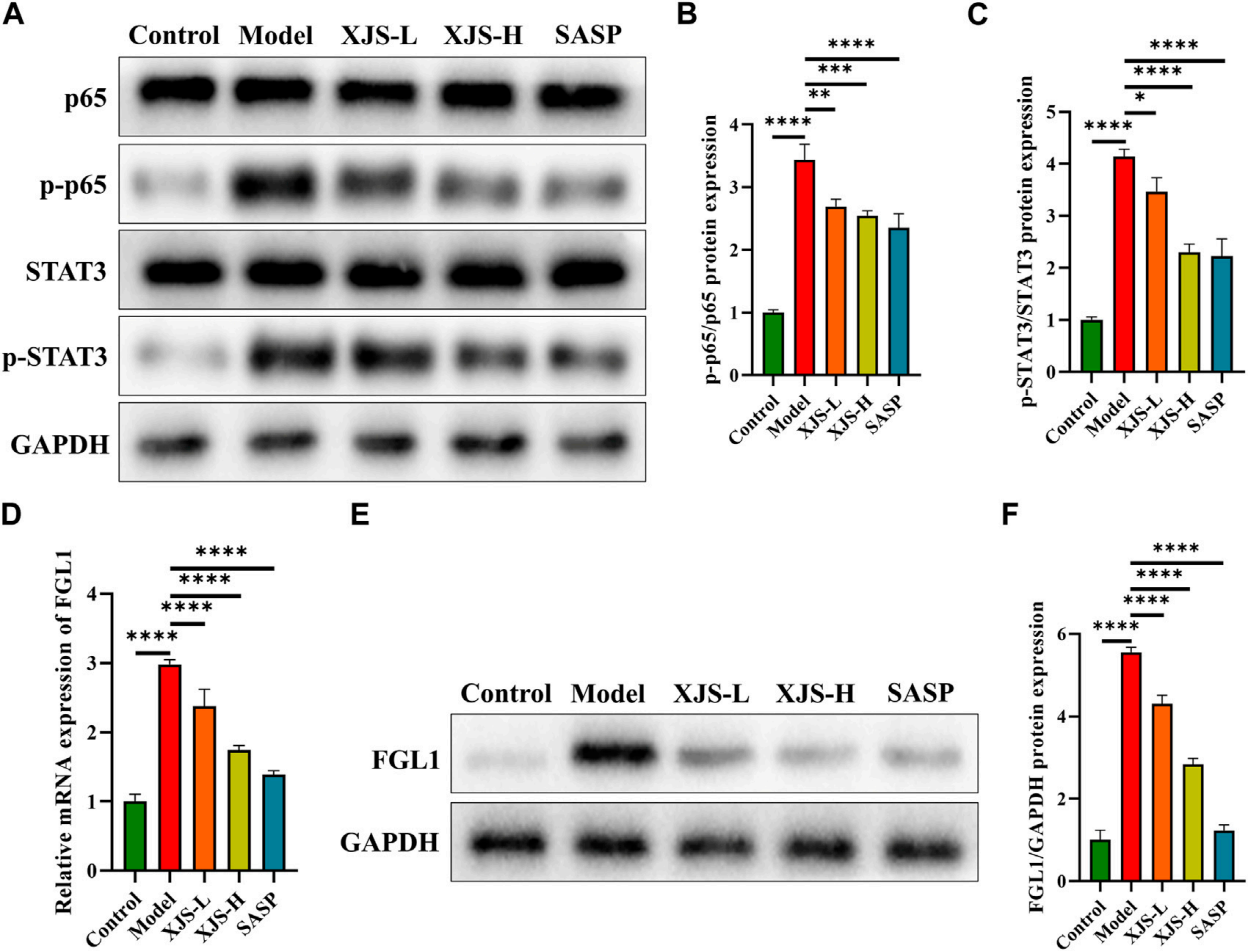
XJS inhibited FGL1/NF-κB/STAT3 positive feedback loop. **(A–C)** The protein expressions of p65 and STAT3 (*n* = 3). **(D)** The mRNA expressions of FGL1 (*n* = 6). (**E, F)** The protein expressions of FGL1 (*n* = 3). ^*^ *p* < 0.05, ^**^ *p* < 0.01, ^***^ *p* < 0.001, ^****^ *p* < 0.0001.

NF-κB and STAT3 are two important transcription factors participating in the initiation and development of CD-related inflammatory response and the regulation of ferroptosis. Therefore, the levels of NF-κB (p65) and STAT3 were examined. Following colitis induction, the protein expressions of p-p65 and p-STAT3, active forms of p65 and STAT3, were significantly upregulated. XJS and SASP treatment inhibited the protein expressions of p-p65 and p-STAT3 (Figures 7A–C). STAT3 can positively regulate the expression of FGL1 that is able to activate p65 (Cheng et al., 2018; Sun et al., 2021). Thus, the FGL1 expressions were detected. Compared to the control group, the model group exhibited an increase in the mRNA and protein levels of FGL1, which were reversed by XJS and SASP administration (Figures 7D–F).

## Discussion

At present, drugs approved for CD treatment have some limitations, including primary non-response, secondary loss response, and severe adverse events. The roles of herbs on treating CD have been emphasized (Torres et al., 2019). XJS, containing Dragon’s blood and Myrrh, is efficacy on promoting intestinal mucosa healing of CD patients (Xu et al., 2022). It has been demonstrated that Dragon’s blood can ameliorate acute ulcerative colitis, another subtype of IBD, by enhancing ribosome synthesis (Lin et al., 2020). Myrrh also has the ability of alleviating experimental colitis (Fatani et al., 2016). However, the underlying mechanism of XJS on treating CD remains obscure. Results in the present study confirmed that XJS might mitigate CD-like experimental colitis through inhibiting ferroptosis in IECs via downregulation of FGL1/NF-κB/STAT3 positive feedback loop signals.

In CD patients, intestinal epithelial barrier function is destroyed, which breaks the homeostasis between luminal contents and intestinal mucosa (Ramos and Papadakis, 2019). Damage of epithelial barrier function may be a trigger of CD onset. Given the protective effect of Dragon’s blood and Myrrh against intestinal epithelial barrier defect, ultrastructure changes in IECs were detected by TEM in this study (Rosenthal et al., 2017; Li et al., 2021). The findings showed shrunken mitochondria and reduction or absence of mitochondrial spine in IECs, which indicated the occurrence of ferroptosis. The integrity of intestinal epithelial barrier can be disturbed by excessive programmed cell death, such as apoptosis, pyroptosis and necrosis. Ferroptosis, as a novel form of programmed cell death, is involved in CD (Xu et al., 2021). Targeting excessive ferroptosis may be an emerging approach for CD treatment (Huang et al., 2022). XJS treatment reduced the amounts of shrunken mitochondria, exhibiting the action of a ferroptotic inhibitor. Meanwhile, XJS treatment improved the TNBS-induced damages of the intestinal mucosa and downregulated the levels of pro-inflammatory cytokines in the colon tissues. These results suggested that XJS might alleviate experimental colitis by inhibition of ferroptosis.

Ferroptosis is a kind of iron- and LPO-dependent cell death. Excessive intake of iron increases a risk of IBD development (Kobayashi et al., 2019). Iron as a redox-active metal participates in the formation of hydroxyl radicals via Fenton reaction. Hydroxyl radical is the most chemically reactive species of activated oxygen, driving the initiation of non-enzymatic LPO (Hassannia et al., 2019). Therefore, iron overload is an important element for the initiation of ferroptotic cell death. In this study, iron concentrations in the colon tissues of CD rats markedly increased, which were downregulated by XJS. To elucidate the molecular mechanism of XJS on suppressing iron overload, the expressions of FPN, FTH and FTL were examined, which regulate the intracellular iron homeostasis. FPN, also known as solute carrier family 40 member 1 (SLC40A1), is the only identified intracellular iron exporter. FPN-mediated transmembrane iron export prevents intracellular iron accumulation (Fang et al., 2021). In non-anemic CD patients, the levels of intestinal FPN protein are lower than those of the healthy controls (Burpee et al., 2011). In our study, downregulated levels of FPN in the colon tissues of CD rats were firstly confirmed. XJS treatment upregulated the FPN expression to promote the transmembrane export of intracellular iron and thereby decrease iron accumulation.

FTH and FTL are two subunits of ferritin, a critical iron storage protein. Although ferritin has been demonstrated to be associated with a decreased risk of IBD onset, its levels are downregulated in IBD patients (Widbom et al., 2020). FTH has the ferroxidase activity to oxidate redox-active ferrous iron into redox-inactive ferric iron, and stores excess intracellular iron in ferritin nanocages (Hu et al., 2021). Several studies have identified that FTH renders cells resist to ferroptosis (Fang et al., 2020; Hu et al., 2021; Lu et al., 2021). However, the expressions of FTH in the colon tissues of experimental colitis mice remain controversial. Some studies have confirmed an increase of FTH expressions in the colitis mice, while decreased FTH expressions are observed in other literatures (Chen et al., 2020; Xu et al., 2020; Chen et al., 2021; Xu et al., 2021; Wu et al., 2023). In this study, FTH levels were downregulated in the colon tissues of the colitis rats, which were reversed by XJS. FTL can stabilize ferritin structure to fulfil its iron storage function. Thus, FTL is considered as an anti-ferroptotic factor (Tang and Kroemer, 2020; Ke et al., 2022). Nevertheless, similar to FTH, FTL expression in experimental colitis and its action to regulate ferroptosis are also controversial (Xu et al., 2020; Chen et al., 2021; Liu et al., 2022). In the present study, elevated FTL expressions were detected in the colon tissues of the colitis rats, which were downregulated after XJS treatment. In fact, the ratio of FTH to FTL determines the capacity of iron storage (Baier et al., 2022). A decreased FTH/FTL ratio results in an attenuated capacity of iron storage and increases intracellular iron accumulation. Furthermore, the FTH/FTL ratio was analyzed in the current study. Results showed a reduced FTH/FTL ratio after TNBS challenge. XJS treatment elevated the ratio to increase the storage of intracellular iron.

The antioxidant activity of FTH can prevent iron-mediated ROS production (Lu et al., 2021). Excess ROS propagates LPO reaction by attacking biomembranes and induces ferroptotic cell death (Su et al., 2019). Thus, the ROS levels in the colon tissues were examined in our study. The results found elevated levels of ROS in the colon tissues of the CD rats, which were accompanied by the accumulation of LPO products, including MDA and 4-HNE. XJS treatment reduced the levels of ROS, MDA and 4-HNE, which indicated the effect of XJS on inhibiting LPO reaction.

To explain the mechanism of XJS on preventing LPO reaction, the SLC7A11/GSH/GPX4 antioxidant axis was detected. GPX4 can neutralize lipid peroxides to protect plasma membranes against peroxidation (Su et al., 2019). In addition, upregulation of GPX4 can reduce ROS production (Kinowaki et al., 2018). Therefore, GPX4 is considered as a core antioxidant enzyme to eliminate ferroptosis. The activity of GPX4 is impaired in IECs of CD patients, which exhibits a sign of LPO (Mayr et al., 2020). Induction of GPX4 can mitigate experimental colitis by suppression of ferroptosis (Wang et al., 2020). GPX4 exerting the antioxidant function needs to consume GSH. GPX4 reduces phospholipid hydroperoxide to its corresponding alcohol phospholipid hydroxide by utilizing two molecules of GSH (Conrad et al., 2016). Depletion of GSH indirectly inactivates the antioxidant activity of GPX4. GSH biosynthesis needs cysteine, glutamate and glycine as substrates. Intracellular cysteine concentration is limited by cystine reduction reaction. Import of extracellular cystine is mediated by SLC7A11, a cystine-glutamate antiporter. Thus, SLC7A11 indirectly affects GSH production. A decreased level of SLC7A11 has been found in IBD patients based on a database analysis (Huang et al., 2022). Upregulation of SLC7A11 to inhibit ferroptosis may be an effective approach for amelioration of colitis (Zhang et al., 2022). We uncovered the inhibition of the SLC7A11/GSH/GPX4 antioxidant system in the colitis rats, of which the activity was enhanced by XJS treatment. XJS might activate this antioxidant axis to inhibit LPO.

NF-κB is a core transcription factor for induction of inflammatory response and intestinal tissue damage by transcriptionally regulating the expressions of multiple pro-inflammatory cytokines like IL-1, IL-6 and TNF-α. Hyperactivation of NF-κB has been found in IECs and macrophages of IBD patients, which is positively associated with the severity of mucosal inflammation (Atreya et al., 2008). STAT3 is another widely expressed cytoplasmic transcription factor, which correlates with CD susceptibility in Chinese Han population (Wang et al., 2014). Overexpressed STAT3 has also been detected in the intestinal mucosa of IBD patients (Vavricka et al., 2018). Inhibition of overexpressed NF-κB and STAT3 can effectively alleviate experimental colitis (Sun et al., 2020; Zhao et al., 2021; Zhang et al., 2022). Recent literature has confirmed the role of NF-κB and STAT3 on regulating ferroptosis (Li et al., 2021; Zhang et al., 2022). Study on NF-κB- and STAT3-mediated ferroptosis involving in the occurrence and development of IBD is scarce. A previous study has demonstrated that knockout of NF-κB in IECs of colitis mice induces endoplasmic reticulum stress-mediated ferroptosis (Xu et al., 2020). This result indirectly highlights the physiological function of NF-κB, but the impact of hyperactivated NF-κB on ferroptosis in colitis needs to be further studied. Another study shows that deficiency of STAT3 enhances ferroptosis in hydrogen peroxide-treated IECs (Huang et al., 2022). This outcome is not verified *in vivo* and does not confirm the impact of hyperactivated STAT3 on ferroptosis in colitis. Our experimental results revealed that overactivation of NF-κB and STAT3 was positively associated with ferroptosis in IECs. Nowadays, the mechanisms of NF-κB and STAT3 on regulating ferroptosis are controversial. Activated NF-κB inhibits ferroptosis in granulosa cells of polycystic ovary syndrome by upregulating the expression of GPX4, but which induces ferroptosis in glioblastoma by repression of SLC7A11 (Li et al., 2021; Tan et al., 2022). Similar to NF-κB, phosphorylated STAT3 not only inhibits ferroptosis in intestinal ischemia/reperfusion-induced acute lung injury by positively regulating SLC7A11, but also induces ferroptosis in hypertensive mice by inactivating the SLC7A11/GPX4 signaling (Qiang et al., 2020; Zhang et al., 2022). According to the available evidences and abovementioned results, overactivated NF-κB and STAT3 might facilitate ferroptosis in IECs via respectively suppressing the expressions of SLC7A11 and GPX4.

FGL1, as a potential immune checkpoint target, has been widely studied in oncology (Qian et al., 2021). In addition, FGL1 has been found to be involved in obesity, lung fibrosis, preeclampsia, diabetes mellitus, non-alcoholic fatty liver disease, and hyperlipidemia (Wu et al., 2013; Li et al., 2016; Jin et al., 2019; Kang et al., 2020; Wu et al., 2020; Cheng et al., 2021). Recently, accumulative evidences indicate that FGL1 can regulate inflammatory diseases. Elevated FGL1 expression is positively correlated with the severity of acute pancreatitis, the disease activity of rheumatoid arthritis, and the severity of radiation-induced acute liver injury (Han et al., 2019; Liu et al., 2020; Sui et al., 2022). Nevertheless, a decreased level of FGL1 is detected in the serum of psoriasis patients (Sun et al., 2022). Above evidences show a controversial role of FGL1 in inflammatory diseases. Deficiency of FGL1 can induce spontaneous dermatitis in gene knockout mice, and supplement of FGL1 recombinant protein improves collagen-induced arthritis (Wang et al., 2019; Lin et al., 2021). These evidences verify the function of FGL1 regulating autoimmunity and immune homeostasis via binding to lymphocyte-activation gene 3 (LAG-3) to inhibit T cell activation (Wang et al., 2019; Lin et al., 2021). However, on the other hand, inhibition of LAG-3 initiates intestinal inflammation by disturbing the immunosuppressive function of Treg cells (Bauché et al., 2018). Nowadays, the impact of FGL1 overactivation on inflammatory process is unclear. Additionally, the role of FGL1 in CD onset and development is unknown. Our previous study has found increased FGL1 levels in both intestinal mucosa and blood plasma of CD patients (Sun et al., 2021). Overexpression of FGL1 enhances lipopolysaccharide-induced inflammatory response in IECs by activating NF-κB signaling (Sun et al., 2021). Results in the present study showed an increase in FGL1 expression after TNBS challenge, which was downregulated following XJS treatment. Overall, XJS might alleviate experimental colitis via suppression of the FGL1/NF-κB/STAT3 positive feedback loop.

It is the strength of this study that the roles of overactivated NF-κB and STAT3 on inducing ferroptosis in IECs and elevated FGL1 on enhancing colitis are uncovered. We also recognize a limitation that the mechanisms of NF-κB and STAT3 on regulating the SLC7A11/GSH/GPX4 antioxidant axis and XJS targeting FGL1 to inhibit colitis are not verified *in vitro*, which will be conducted in a future study.

## Conclusion

**FIGURE 8:**
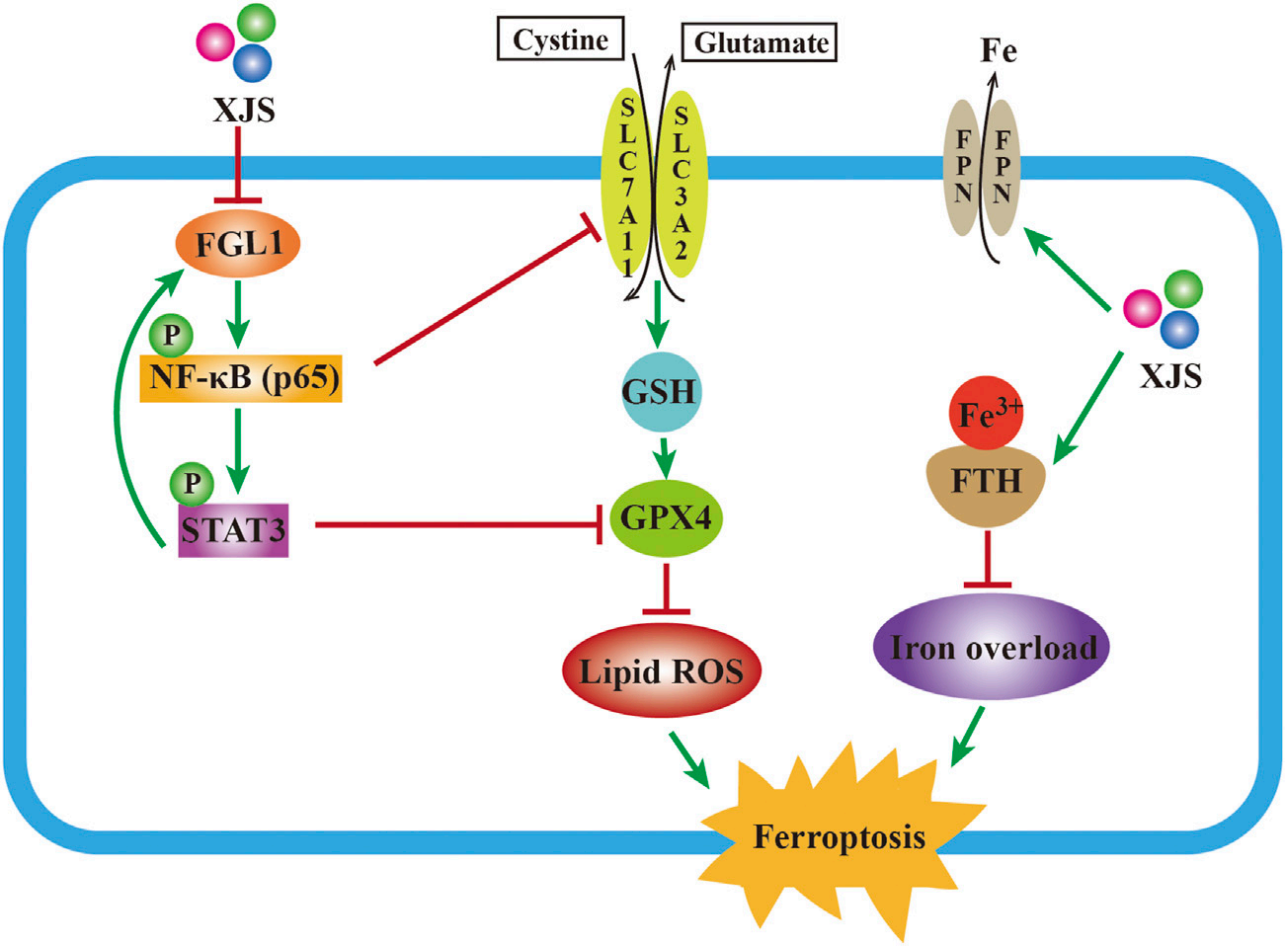
Mechanism schematic of XJS alleviating colitis by inhibition of ferroptosis.

The present study displayed that XJS alleviated CD-like experimental colitis via inhibition of ferroptosis in IECs. Mechanistically, XJS inhibited iron overload by increasing the levels of FPN and FTH, and suppressed LPO through enhancement of the SLC7A11/GSH/GPX4 antioxidant system by repressing the FGL1/NF-κB/STAT3 positive feedback loop (Figure 8). This work might provide new targets and drugs for CD treatment.

## Data Availability

The original contributions presented in the study are included in the article/Supplementary Material, further inquiries can be directed to the corresponding author.
